# Evaluation of the virucidal effects of rosmarinic acid against enterovirus 71 infection via in vitro and in vivo study

**DOI:** 10.1186/s12985-019-1203-z

**Published:** 2019-07-31

**Authors:** Wen-Yu Lin, Yu-Jen Yu, Tzyy-Rong Jinn

**Affiliations:** School of Chinese Medicine, China Medical University, Taichung, 40402 Taiwan, Republic of China

**Keywords:** Enterovirus 71, Herbal medicine, Molecular docking, Neonatal mice, Rosmarinic acid, Virucidal efficacy

## Abstract

**Background:**

Although enterovirus 71 (EV71) is an important public health threat, especially in the Asia-Pacific region, there are still no effective drugs or vaccines to treat and prevent EV71 infection. Therefore, it is critical to develop prophylactic and therapeutic agents against EV71. Rosmarinic acid (RA), a phytochemical, has been discovered to possess a broad spectrum of biological activities.

**Methods:**

The virucidal effects of RA on EV71 were determined by MTT, western blot, median cell culture infectious dose, apoptosis detection, plaque reduction, semi-quantitative real-time polymerase chain reaction, immunofluorescence detection, molecular docking analysis, and mouse protection assay.

**Results:**

RA showed a strong protective effect against EV71 infection in human rhabdomyosarcoma cells when the multiplicity of infection was 1, with a low IC_50_ value (4.33 ± 0.18 μM) and high therapeutic index (340). RA not only protected cells from EV71-induced cytopathic effects, but also from EV71-induced apoptosis. The results of time-of-addition analysis demonstrated that the inhibitory activity of RA was highest at the early stage of viral infection. Consistent with this, the infectivity of EV71 in the early stage of viral infection also was observed to be limited in neonatal mice treated with RA. Further, molecular docking predicts that RA could replace the natural pocket factor within the VP1 capsid-binding hydrophobic pocket.

**Conclusions:**

This study suggests that RA has the potential to be developed as an antiviral agent against initial EV71 infection to prevent or reduce EV71-induced pathogenesis and complications, since RA can effectively reduce EV71 infection in the early stages of viral infection.

## Background

Hand, foot, and mouth disease (HFMD) induced by enterovirus 71 (EV71) is a critical public health threat, especially in the Asia-Pacific region [1]. EV71 belongs to the *Enterovirus* genus within the *Picornaviridae* family. Its genome is a single-stranded, positive-sense RNA with approximately 7411 nucleotides and is enclosed in an icosahedral capsid composed of four structural proteins, VP1–VP4 [1, 2]. The EV71 viral RNA genome does not have proofreading activity for faithfully replicating viral RNA; as such, mutations are frequently generated during its replication [2], resulting in many variants and drug-resistant mutant strains which make the treatment of EV71 infection complex and difficult [1, 2]. Therefore, in addition to the development of anti-EV71 agents for treating EV71 infection, the development of effective antiviral prophylaxis and synergistic reagents is also critical to the prevention and treatment of drug-resistant EV71 variants.

Some phytochemicals extracted from different medicinal herbs have been shown to inhibit EV71 infection in cultured cells, such as oblongifolins J and M, gallic acid, polydatin, resveratrol, aurintricarboxylic acid, kaempferol, baicalin, and apigenin [3–5]. Further, punicalagin [6] and chebulagic acid [7] have been found to inhibit EV7 infection both in cultured cells and in mouse models. Our previous in vitro study described two potent antiviral compounds, magnesium lithospermate B (MLB) and rosmarinic acid (RA), which are responsible for the anti-EV71 activity of *Salvia miltiorrhiza* [8]. The latter is more likely to be developed as a therapeutic or prophylactic agent against EV71 infections for economic reasons as well as convenience; RA is cheaper and easier to extract and synthesize for clinical use than MLB [9, 10].

RA is a naturally occurring phenolic ester derived from caffeic acid and (R)-(+)-3-(3,4)-dihydroxyphenylacetic acid [9]. It is found in many plant species, including rosemary (*Rosmarinus officinalis* L.), spearmint (*Mentha* spp.), lemon balm (*Melissa officinalis* L.), *Perilla frutescens* (L.) Britton, *Rabdosia rubescens* (Hemsl.) *H. hara*, and *S. miltiorrhiza* Bunge [9–11]. Importantly, it exhibits many significant biological and pharmacological activities relating to human health in clinical studies. These include anti-inflammatory, antioxidant, antiangiogenic, antitumor, antiviral, antimicrobial, and neuroprotective functions [8–13]. Together, the results of previous reports suggest that RA is safe and ideal for use in medical applications. Thus, in the present study, we performed a detailed investigation of the antiviral efficacy of RA against the C4 subgenotype of EV71 (EV71-C4) both in vitro using human rhabdomyosarcoma (RD) cells and in vivo using neonatal mice. The C4 subgenotype of EV71 is highly prevalent in parts of Asia, including Cambodia, China, Taiwan, Thailand, and Vietnam [1]. For simplicity, EV71-C4 (Genebank No. HM807310) is referred to in the present article as “EV71.”

## Methods

### Cell culture and virus

Human rhabdomyosarcoma (RD; ATCC accession no. CCL-136) cells were grown in Dulbecco’s modified Eagle’s medium (DMEM, Invitrogen) supplemented with 10% fetal bovine serum (FBS, HyClone) and 1% non-essential amino acids (Thermo Fisher) at 37 °C in an incubator containing 5% CO_2_. Enterovirus 71 (EV71; Genbank No. HM807310) was kindly provided by the clinical virology laboratory of China Medical University Hospital (Taichung, Taiwan) and was propagated in the RD cells. The EV71 used in the present study belongs to the C4 subgenotype, as verified through sequence analysis of the VP1 region [14]. The virus titer was quantified as plaque-forming units per milliliter (PFU/mL).

### Infection assays

For virus infection assay, RD cells (6 × 10^4^ cells in 1 mL medium/well) were seeded in 24-well plates and incubated overnight. Then, the monolayer RD cells were infected with EV71 and simultaneously treated with different concentrations of RA (Sigma-Aldrich, USA). RA dissolved in sterile PBS and the final concentration of PBS did not exceed 0.5% in the treated culture medium. At different time points after infection (hours post infection, h.p.i.), the EV71-induced cytopathic effect (CPE) was observed under a microscope and quantitatively measured by MTT assay (Sigma, St. Louis, MO, USA). Viral infection, MTT, plaque reduction, and western blot analyses were carried out according to methods described previously [8]. A western blot was performed using anti-VP-1 antibody (1:10,000; Abnova, Taiwan) and β-actin antibody (1:10,000; Santa Cruz, California) as primary antibodies. Horseradish peroxidase (HRP)-conjugated anti-mouse IgG antibody (1:10,000; Cell Signaling, USA) was used as a secondary antibody, and the protein bands were digitized using an Alphamager image-analyzing system with ImageJ software (Alpha Innotech Corporation, San Leandro, CA).

### Apoptosis assay

For the apoptosis assay, RD cells were plated overnight (6 × 10^4^ cells per well). Afterwards, the cells were infected with EV71 at a multiplicity of infection (MOI) of 1 and simultaneously incubated with or without 10 μM RA for 1 h at 37 °C. After 1 h, the cells were washed three times with PBS and incubated for another 24 h in fresh 10% FBS medium. Subsequently, RD cells were transferred to a glass tube. Then, using the FITC-Annexin V Apoptosis Detection Kit I (Cat No.556547; BD Biosciences, San Jose, California, USA), 5 μL FITC-conjugated annexin V and 5 μL propidium iodide (PI) were added, followed by incubation for 15 min at room temperature in the dark. The stained apoptotic and necrotic cells were analyzed by flow cytometry (FACScan, USA) [15].

### Immunofluorescence assay

For the immunofluorescence assay, 1 × 10^4^ RD cells were grown overnight on coverslips coated with poly-L-lysine (1 mg/mL), then treated with a final concentration of 10 μM/mL RA while simultaneously being infected with EV71 at a MOI of 1 for 1 h. Subsequently, cells were fixed with 4% paraformaldehyde and permeabilized with 0.5% Triton X-100 (in PBS) for 15 min, then blocked with 1% BSA for 2 h at room temperature. Afterwards, the cells were incubated for 60 min at 37 °C with primary anti-EV71 antibody using the anti-VP0/VP2 monoclonal antibody MAB979 (Millipore, USA) [16, 17]. Cells were then washed three times with PBS at room temperature for 10 min, followed by incubation with a secondary antibody (Alexa Fluor 488 anti-mouse IgG; Invitrogen, USA) labeled with fluorescein isothiocyanate (FITC). Cell nuclei were stained with 4,6-diamidino-2-phenylindole (DAPI) (Sigma, St. Louis, MO) for 5 min. After washing twice with PBS, the coverslips were mounted in glycerol and observed under an inverted fluorescence microscope (Olympus IX71, Japan). Fluorescence intensity was quantified and analyzed using Image J software (NIH, Bethesda, MD).

### Secondary infection assay

RD cells (6 × 10^4^ cells/well) were seeded on a 24-well plate and incubated overnight. As previously described, the cells were infected with EV71 at a MOI of 1 and simultaneously treated with different concentrations of RA for 24 h. At 24 h.p.i., supernatants were collected from the treated cultures and freeze-thawed to release the infectious virus. Afterward, the samples were titrated by the CCID_50_ method and the Kärber formula was applied (WHO, 1997).

### Semi-quantitative real-time polymerase chain reaction (SqRT-PCR)

Total RNA was extracted from cells using a Total RNA Purification Kit (GMbiolab, Taiwan) according to the manufacturer’s protocols, and RNA concentration was assessed based on *A*_260_ with a UV-Vis spectrophotometer (TU1901, Persee, Beijing, China). The number of copies of the viral genome was determined by real-time PCR. Then, cDNA was synthesized using the RevertAid™ First Strand cDNA Synthesis Kit (Thermo Fisher). About 1.5 μg total RNA was reverse transcribed into cDNA using random hexamer primers according to the manufacturer’s instructions. For real-time PCR, 20 μL reaction mixtures were prepared containing 2 μL cDNA (equivalent to ~ 100 ng reverse transcribed RNA), 400 nM primers, and 10 μL Power SYBR™ Green PCR Master Mix (Thermo Fisher). The EV71 VP1 region was amplified with EV-qF (5′-GCGTGTCCTAACAACATGATGGGCAC-3′) and EV-VP1R (5′-CGGTTAACCACTCTAAAGTTGCCCAC-3′) primers and determined by the online Enterovirus Genotyping Tool, version 0.1 (http://www.rivm.nl/mpf/typingtool/enterovirus/) [18]. The internal control GAPDH region was amplified with GAPDH-F (5′-CAGACACCATGGGGAAGGTGAAG-3′) and GAPDH-R (5′-TGAAGGGTCATTGATGGCAAC-3′) primers [19]. Reactions were run on a StepOnePlus™ Real-Time PCR System (Thermo Fisher). The cycling conditions were 10 min at 95 °C, followed by 40 cycles of 95 °C for 15 s, then 60 °C for 1 min. The results were analyzed using the comparative delta-ct method in StepOne version 2.2.2 (Thermo Fisher). The ratio of viral RNA (EV71 only) to the internal control was normalized and arbitrarily set to 1, which was defined as 100%.

### Molecular modeling

An X-ray crystal structure of human EV71 mature virus [20, 21] at a resolution of 3.0 Å (PDB code 3VBS) was obtained from a protein data bank (http://www.rcsb.org) [22] for use as a modeling template using Discovery Studio version 4.1 (DS4.1; Accelrys Software Inc.). The 3D structure of RA (CID: 5281792) used in this study was retrieved from the PubChem database (https://pubchem.ncbi.nlm.nih.gov) and the three-dimensional homology model of EV71 VP1 from ADN52609.1 (Human EV71 strain cmuh-050530-5) was constructed using MODELER and further refined using CHARMM in DS4.1. In order to facilitate the docking process, the structures of modeled VP1 and RA were minimized by applying the DS4.1 CHARMM force field method. Molecular docking was performed with RA compound into the refined model VP1 by employing the DS4.1 CDOCKER tool. After RA was docked into the binding site of the model VP1, the binding energies for the generated conformations were calculated to select the best docked molecule. The hydrophobic and hydrogen bond interactions between the VP1 and RA were generated in LigPlot [23].

### Mouse protection assay

For the mouse protection assay, 3-day-old wild-type ICR mice (BLTW: CD1 strain) were acquired from BioLasco Taiwan Co., Ltd. and divided into three groups: A, B, and C (*n* = 10 per group). Mice in group A (EV71 only) were intraperitoneally injected with EV71 (1 × 10^6^ CCID_50_, lethal dose [24]). Mice in group B were intraperitoneally co-injected with both infected EV71 (1 × 10^6^ CCID_50_) and treated RA (20 mg/kg). Group C mice were intraperitoneally injected with RA (20 mg/kg) as a mock control. The mice in all three groups were treated once daily for 3 d and monitored daily until 6 d after inoculation. The survival rates and body weights of the treated mice were then analyzed to assess the effects of RA against EV71 infection in vivo.

### Statistical analysis

All statistical analyses were performed and evaluated by one-way ANOVA using JMP 5.01 software (JMP, 2007, by SAS Institute). A *P* value < 0.05 was considered statistically significant. Data were obtained from three separate experiments and are presented as the mean ± S.D.

## Results

### Analysis of the virucidal efficacy of RA against EV71

**Fig. 1:**
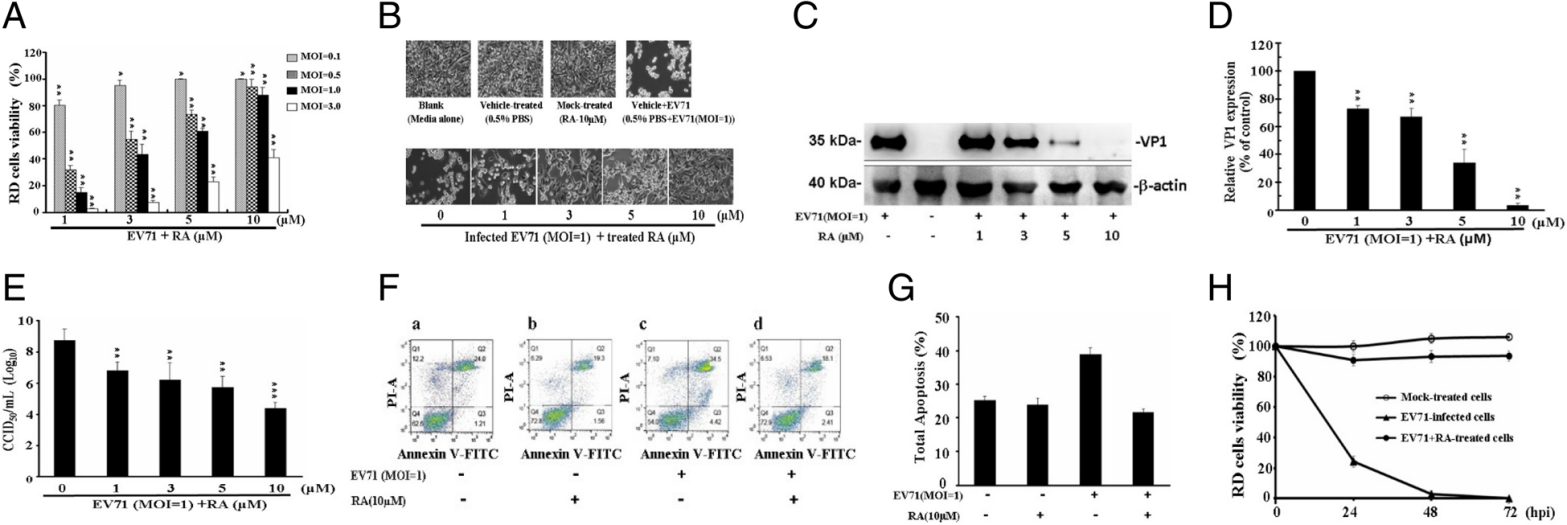
Analysis of the virucidal effects of rosmarinic acid (RA) on EV71 infection. **a** RD cells (6 × 10^4^) were infected with EV71 at multiplicity of infections (MOIs) of 0.1, 0.5, 1, and 3 and treated simultaneously with 1, 3, 5, or 10 μM RA for 24 h. Cell viability was determined by MTT assay. Percentage viability was calculated as (OD_(infected + treated)–_OD_infected_)/ (OD_infected_) × 100. **b** RD cells (6 × 10^4^) were infected with EV71 virus at a MOI of 1 and treated simultaneously with or without RA. At 24 h post infection, the cytopathic effect was observed under a phase-contrast microscope (100× magnification). **c** Western blot analysis. RD cells (6 × 10^4^) were infected with EV71and treated simultaneously with or without RA for 24 h at 37 °C. Cells were collected and the intracellular viral protein VP1 was detected by western blotting. β-actin was used as the loading control. **d** VP1 levels relative to β-actin were calculated. Cells treated with EV71 virus only were used as the positive control (100% reference). **e** Median cell culture infectious dose (CCID_50_). EV71 (MOI = 1; 6 × 10^4^ PFU) was mixed with 0, 1, 3, 5, or 10 μM RA for 24 h at 37 °C. Supernatants were immediately added to RD cells to obtain the CCID_50_. The viral titer is presented as CCID_50_/mL (log_10_ EV71 titer). **f** Effects of RA on EV71-induced apoptosis. EV71-induced apoptosis was detected by flow cytometry following annexin V-FITC/PI double staining, and analyzed with Cell Quest software. Scatter diagram: Q2 = late apoptosis; Q3 = early apoptosis. **g** Total apoptosis (Q2 + Q3) is shown. Triplicate wells were used for each treatment. All results were obtained from three separate experiments and are presented as means ± S.D. **h** Time-dependent efficacy of RA against EV71 infection. Cells (6 × 10^4^) were collected 24, 48, and 72 h post infection. Viability was assessed by MTT assay with blank cells (in DMEM with 10% FBS alone) set to 100%. In this study, blank control was uninfected-EV71 and no compounds in the DMEM-10%FBS media. Vehicle control was 0.5% PBS in DMEM-10%FBS media. Mock-treated control was treated-10 μM of RA in DMEM-10%FBS media. Infected control was infected-EV71 alone in DMEM-10%FBS media. Data were obtained from three separate experiments and are presented as the mean ± S.D. ^*^P < 0.05, ^**^P < 0.001, ^***^P < 0.0001, as compared with control

To assess the virucidal efficacy of RA, RD cells were infected with EV71 at different MOIs (0.1, 0.5, 1, and 3) while being treated simultaneously with 1, 3, 5, or 10 μM RA for 24 h at 37 °C (Fig. 1a). Cells infected with EV71 alone at MOIs of 0.1, 0.5, 1, and 3 exhibited approximately 17.8, 45.2, 79.8, and 98% cell death, respectively, when compared to blank cells (untreated, uninfected in DMEM with 10% FBS, and normalized to 100% viability) (data not shown). The viability of cells simultaneously infected with EV71 at a MOI of 1 and treated with RA at concentrations of 1, 3, 5, and 10 μM was 14.5% ± 4.01, 43.6% ± 7.43, 60.6% ± 2.32, and 88.0% ± 6.05%, respectively (Fig. 1a). EV71 infection of RD cells was not inhibited by 0.5% PBS (vehicle) (Fig. 1b). Administration of 0.5% PBS and 10 μM RA was not cytotoxic to RD cells, and the viability and morphology of cells treated in this way were almost equal to those of the blank control, in which no compound was added to the DMEM–10% FBS media (Fig. 1b). RD cell death and CPE induced by infection with EV71 were greatly inhibited 24 h.p.i. by treatment with 10 μM RA (Fig. 1a and b). The IC_50_, IC_75_, IC_90_, and therapeutic index values of RA are shown in Table 1. In addition, the viral VP1 protein and progeny virus yield were detected. When infected cells (MOI = 1) were treated with 1, 3, 5, and 10 μM RA, the amount of viral VP1 protein detected was 73% ± 6.2, 67% ± 6.4, 34% ± 10, and 3.6% ± 1.5% that of infected cells not treated with RA, respectively (normalized to 100%) (Fig. 1c and d). Meanwhile, the antiviral activity of RA on the progeny virus yield was also observed. When treated with 1, 3, 5, and 10 μM RA, EV71 production was reduced by log 1.95, 2.56, 3.04, and 4.36, respectively, compared to EV71 (MOI = 1) infection alone (log 8.75) (Fig. 1e).

**Table 1:** Antiviral activity of rosmarinic acid against EV71 infection

MOI	EC_50_ (μM)	EC_70_ (μM)	EC_90_ (μM)	TI
0.1	2.19 ± 0.11	3.42 ± 0.07	4.16 ± 0.15	672.15
0.5	3.76 ± 0.24	5.23 ± 0.11	6.03 ± 0.14	393.33
1.0	4.33 ± 0.18	5.69 ± 0.22	6.50 ± 0.27	340.39

EC_50_, EC_75_, and EC_90_ represent the concentrations that inhibit EV71-induced cytotoxicity by 50, 75, and 90%, respectively. RD cell cytotoxicity was determined by the MTT assay. Data are shown as means ± SD. Therapeutic index (TI) = CC_50_/EC_50_

To further determine whether RA could affect EV71-induced apoptosis, annexin V-FITC/propidium (PI) double staining analysis was performed. Late apoptotic and necrotic cells were identified as FITC(+) and PI(+) (Fig. 1f, Q2) and early apoptotic cells were identified as FITC(+) and PI(−) (Fig. 1f, Q3). Viable cells were identified as FITC(−) and PI(−) (Fig. 1f, Q4). Without any treatment (blank control), 24% of the cells were late apoptotic (Fig. 1f (a)), and the percentage of cells in late apoptosis was approximately to 19.3% when cells were incubated with 10 μM RA alone (Fig. 1f (b)). Late apoptosis induced by EV71 was reduced from 34.5 to 18.1% when RD cells were incubated with 10 μM RA (Fig. 1f (c) and (d)). The overall extent of apoptosis was calculated and is shown in Fig. 1g. Based on these results, RA not only protect cells from EV71-induced CPE, but also potentially protects infected cells from death and apoptosis. It is worth noting that the apparent inhibitory effect of RA on EV71 viral infections lasted up to 72 h.p.i. (Fig. 1h). Cells treated with only 10 μM RA were used as a mock control, in which the cell viability was nearly equal to that of the blank control (Fig. 1h). When RA (10 μM) was administered to EV71-infected cells (MOI = 1) cell viability was approximately 90.9% ± 3.74, 93.3% ± 3.92, and 93.6% ± 2.58% at 24, 48, and 72 h.p.i., respectively. In contrast, cells infected with EV71 without RA had very low viability at 24 and 48 h.p.i., and prolonged incubation (up to 72 h) led to total cell death (Fig. 1h). These results indicate that RA has potent activity against EV71 (MOI = 1) infectivity at a concentration as low as 10 μM.

### Time-of-addition effects of RA on EV71 infection

**Fig. 2:**
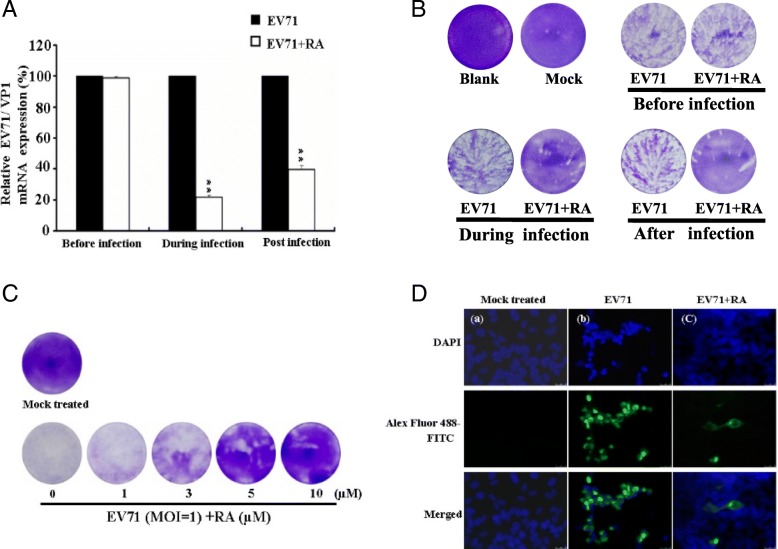
Time-of-addition analysis of rosmarinic acid (RA) against EV71 infection. **a** Viral VP1 mRNA was detected by quantitative real-time polymerase chain reaction. RD cells were treated with 10 μM RA before (− 2 h), during (0 h), or post (+ 2 h) EV71 (MOI = 1; 6 × 10^4^ PFU) infection for 1 h, then viral RNA was extracted. **b** Viable viruses were detected by plaque reduction assay. RD cells were treated with 10 μM RA before (− 2 h), during (0 h), or post (+ 2 h) EV71 (MOI = 1; 6 × 10^4^ PFU) infection for 1 h, then viable viruses were detected by plaque reduction assay. **c** Plaque reduction assay. EV71 (6 × 10^4^ PFU) was mixed with 0, 1, 3, 5, or 10 μM RA for 1 h at 37 °C. Virus-treated and mock control tubes were diluted 20-fold and applied to infect RD cells for 1 h. **d** Immunofluorescence assay. RD cells (1 × 10^4^) were infected with EV71 at a MOI of 1 (1 × 10^4^ PFU) and treated simultaneously with or without 10 μM RA at 37 °C. At 1 h post infection, images were obtained with a fluorescence microscope (100× magnification). Green fluorescence was produced by Alexa Fluor 488-FITC and blue fluorescence was produced by DAPI. Each assay was repeated three times. Data were obtained from three separate experiments and are presented as the mean ± S.D. ^*^P < 0.05, ^**^P < 0.001, as compared with control

To understand the effects of time-of-addition, RA was added to EV71-infected RD cells at different time points, and then SqRT-PCR was performed (Fig. 2a). The results showed that treatment of RD cells with 10 μM RA during (0 h) and post (+ 2 h) EV71 infection (MOI = 1) reduced viral VP1 mRNA to 21.9% ± 1.03 and 39.9% ± 2.33% that detected in cells not treated with RA (Fig. 2a), respectively. However, the addition of RA before (− 2 h) viral infection had no significant inhibitory effect. A plaque reduction assay (Fig. 2b) was also carried out, the results of which were consistent with the results shown in Fig. 2a. The plaque reduction assay (Fig. 2b) was observed and calculated that the inhibitory rates were 5.54 ± 1.7%, 88.6 ± 3.2% and 72.8 ± 3.8% at the before, during or post-EV71 infection, respectively (data not shown). The virucidal activity of RA was further assessed when EV71 (MOI = 1) was directly mixed with different concentrations of RA for 1 h (during viral infection). As shown in Fig. 2c, treatment with 10 μM RA significantly inhibited EV71-induced plaque formation. Meanwhile, an immunofluorescence assay was carried out on cells treated with 10 μM RA while simultaneously being infected with EV71 at a MOI of 1 for 1 h. EV71 capsids were detected by antibody MAB979 (anti-VP0/VP2) and were then stained with Alexa Fluor 488-FITC (green fluorescent). The capsids were clearly visible in cells treated with EV71 alone, but only a small amount of green fluorescence was detected in infected cells when RA (10 μM) was administered during viral infection (Fig. 2d).

### Molecular docking of RA in VP1 of EV71

**Fig. 3:**
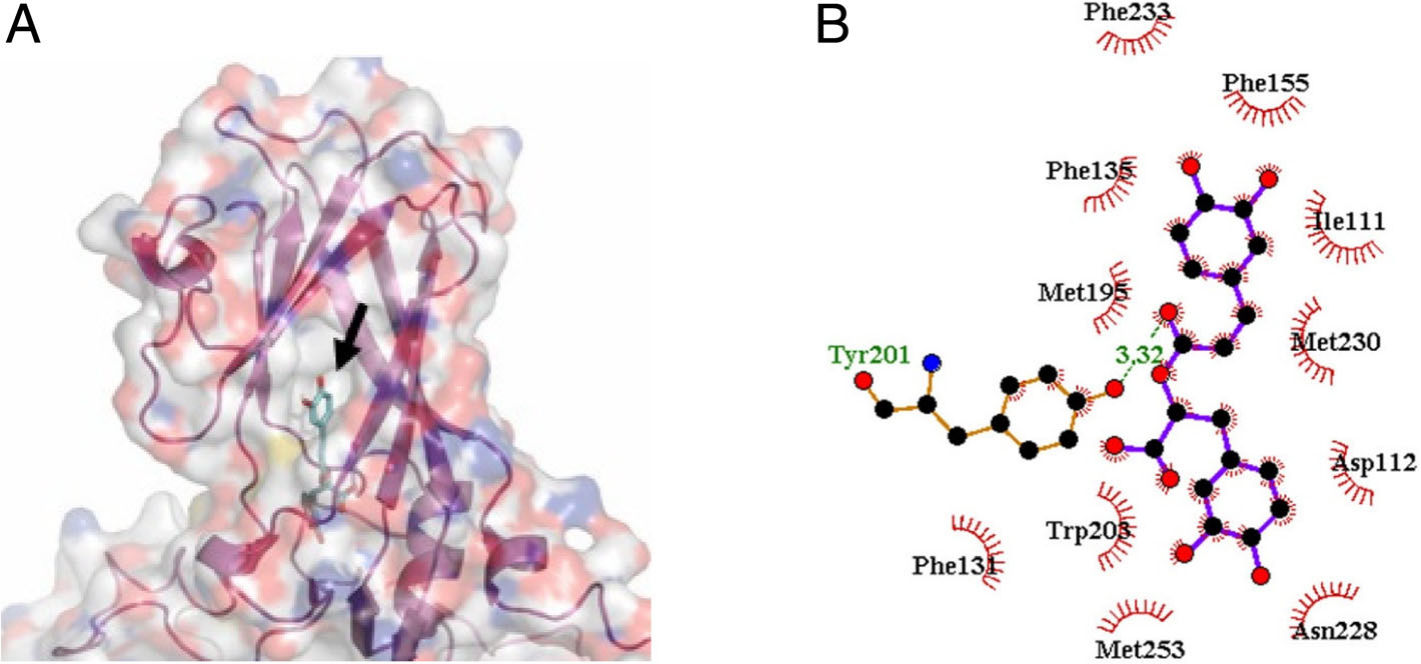
Molecular docking of rosmarinic acid (RA) to the VP1 of EV71. **a** Transparent view of the structure of RA (green) docked in the hydrophobic pocket within VP1 (purple). **b** Two-dimensional representation of hydrophobic interactions and hydrogen bonding within the VP1–RA complex. The side chains of residues forming the hydrophobic interaction are shown as arcs. A hydrogen bond was predicted in the Tyr201 of VP1, as shown by the dashed line. Location of RA is indicated by an arrow

An X-ray crystal structure of the human EV71 mature virus with a resolution of 3.0 Å (PDB code 3VBS) was used as a modeling template [20–22]. Molecular docking indicated that RA binds to the hydrophobic pocket within VP1 (Fig. 3a). As shown in Fig. 3b, RA is bound at the surface of the opening of the hydrophobic pocket within VP1 by hydrophobic interactions. Additionally, the O9’ of RA and Tyr201 (Oη) of VP1 are 3.32 Å apart, which suggests a potential hydrogen bond (indicated by the dashed line) within the VP1–RA complex (Fig. 3b).

### Effects of RA on EV71 infection in neonatal mice

**Fig. 4:**
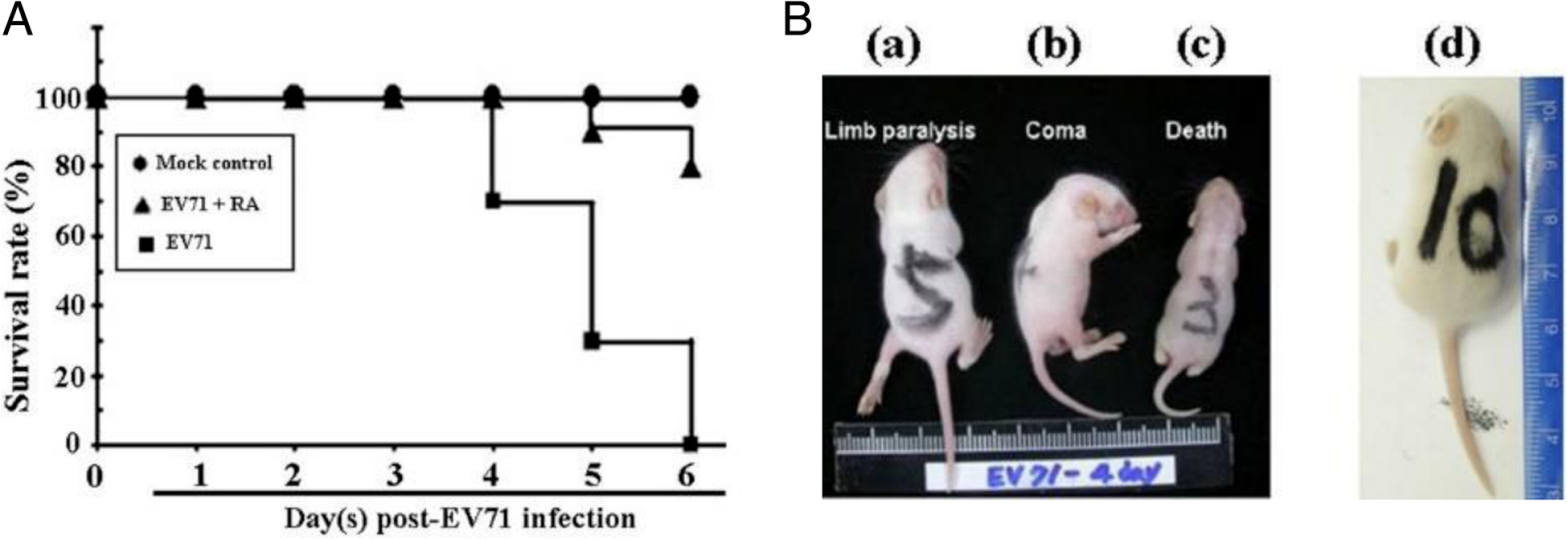
Effects of rosmarinic acid (RA) on EV71 infection in neonatal mice. Three-day-old ICR neonatal mice were inoculated intraperitoneally with EV71 (1 × 10^6^ CCID_50_). **a** Daily survival rate up to 6 d post inoculation. **b** Typical phenotypes of rare hair and paralysis of hind limbs caused by EV71 infection alone (group A; positive treatment) are shown in: (a) the posterior limb of a severely paralyzed mouse; (b) a comatose mouse; and (c) a dead mouse at 4 d post infection. Mice were defined as comatose based on a loss of reflexes and response to pain. All healthy mice in group B (RA + EV71 treatment) and group C (RA treatment only) resembled the representative picture (d) on day 4 post inoculation

Based on the highest inhibitory activity of RA recorded during viral infection, RA was directly incubated with EV71 and then co-injected into mice (rather than infecting mice with EV71 for 1 h before administering RA). This protocol was modified from previously reported animal experiments on EV71 infection [24]. In this experiment, EV71 (1 × 10^6^ CCID_50_)-infected mice (group A) began to develop symptoms of paralysis at 3 d post infection (d.p.i.), and all died within 6 d (survival rate = 0%). However, only two mice in group B died within 6 d (survival rate = 80%); these mice were simultaneously treated with RA (20 mg/kg) and infected EV71 (1 × 10^6^ CCID_50_) (Fig. 4a). No mice died within 6 d in group C, in which mice were treated with 20 mg/kg of RA alone (survival rate = 100%; Fig. 4a). The typical symptoms of sparse hair, paralysis of hind limbs, and death were found in the EV71-infected mice that were not treated with RA (group A). As shown in Fig. 4b, a severely paralyzed mouse, a comatose mouse, and a dead mouse were all clearly observed in group A at 4 d.p.i. However, the symptoms of EV71 infection were prevented and reduced in group B mice treated with RA (Fig. 4d). EV71-induced paralysis and death were noticeably reduced by co-treatment with 20 mg/kg RA (group B). The effect of RA on body weight during EV71 infection (Table 2) was consistent with the survival results (Fig. 4a). As shown in Table 2, despite a slow increase in body weight for the first 2 d, mice treated with both RA and EV71 (group B) and RA only (group C) began to recover 3 d.p.i. In contrast, the weight of mice in the EV71-infected group not treated with RA (group A) did not increase during the infection period. Taken together, the results show that 20 mg/kg RA at the early stage of EV71 infection prolonged survival time and reduced mortality in mice inoculated with a lethal dose of EV71.

**Table 2:** Effects of rosmarinic acid on body weight changes during EV71 infection in neonatal mice

Day(s) post-EV71	Body weights (g)					
infection	1	2	3	4	5	6
Treated EV71	2.55 ± 0.17	2.66 ± 0.17	2.68 ± 0.08	2.69 ± 0.09	2.72 ± 0.15	2.72 ± 0.13
Treated EV71 + RA	2.60 ± 0.12	2.97 ± 0.32	3.37 ± 1.09	3.72 ± 0.94	4.24 ± 1.18	4.51 ± 1.55
Mock control	2.63 ± 0.17	3.02 ± 0.99	3.50 ± 1.45	3.91 ± 1.30	4.59 ± 1.07	4.97 ± 1.24

The average body weight of 3-day-old male ICR mice is 2.46 ± 0.12 g/mouse. Data are shown as means ± SD

## Discussion

In this study, RA provided significant protection to RD cells against EV71-induced CPE, even at concentrations as low as 10 μM; the IC_50_ was 4.33 ± 0.18 μM and the therapeutic index (TI) was 340 when RD cells were infected with EV71 at a MOI of 1 (Table 1). Notably, the TI of RA on EV71 at a MOI of 1 was much higher (> 100 times) than that at a MOI of 3, at 340 and 3.19 [8], respectively. This result indicates that the antiviral efficacy of RA was very high at a MOI of 1, although this was not the case at a MOI of 3 [8]. The infectivity of EV71 significantly declined upon treatment with 10 μM RA at MOI of 1 (Fig. 1a–h). The inhibition of EV71 infectivity by RA was similar to that by MLB in infected RD cells at a MOI of 1 (data not published). In addition, some phytochemical compounds, such as apigenin (TI = 7.67; MOI of 0.03 TCID_50_) [25], resveratrol (TI = 4.01 at a MOI of 2) [26], punicalagin (TI = 20 at 100 TCID_50_) [6], chebulagic acid (TI = 16 at 100 TCID_50_) [7], were studied in RD cells, and oblongifolia J (TI = 1.5 at 100 TCID_50_) [27], oblongifolia M (TI = 2.4 at 100 TCID_50_) [27], and gallic acid (TI = 99.57; 100 TCID_50_) [28] were observed in Vero cells. RA exhibited the potential to inhibit EV71 activity; the TI of RA was 340 at a MOI of 1 (IC_50_ = 4.33 ± 0.18 μM) in the present study and 3.19 at a MOI of 3 in our previous report [8]. Notably, RA limited EV71 induced CPE (Fig. 1b) and apoptosis (Fig. 1g), and its effects persisted until 72 h.p.i. (Fig. 1h). Thus, RA could prevent or reduce EV71-induced pathogenesis and complications since EV71-induced apoptosis is a crucial mechanism in the spread of viral progeny and is associated with HFMD pathogenesis [1].

To evaluate its mechanism of action, RA was administered to infected RD cells at different time points. Results showed that RA inhibited EV71 infection more efficiently when it was present continuously during virus–cell adsorption than if added before adsorption (Fig. 2a and b), as is the case for pleconaril/pirodavir-like anti-EV71 compounds [29]. The highest inhibitory activity of RA occurred during viral infection, at the early stage of EV71 infection (Fig. 2a and b). The infectivity of EV71 was directly reduced by treatment with RA during viral infection in a concentration-dependent manner (Fig. 2c). RA was also found to greatly reduce or disrupt the ability of EV71 to infect cells (Fig. 2d). Based on these results, the antiviral activity of RA may occur via the disruption of viral particles, which affects their ability to infect cells, reducing the infectivity of EV71 (Fig. 2c and d). The docking results predicted that RA binds in the VP1 capsid-binding hydrophobic pocket (Fig. 3a and b). The predicted binding pattern of the RA–VP1 complex was the same as other inhibitors of EV71 [3-(4-pyridyl)-2-imidazolidinone derivatives], such as WIN 51711, ALD, and NLD, which bind in the binding groove located in the VP1 canyon [30–32]. The predicted RA molecule binds to the aromatic amino acids Phe135 and Phe155 through hydrophobic interactions in the VP1 binding groove (Fig. 3b), as do capsid-binding inhibitors of EV71 [30–32]. To our knowledge, this is the first report of RA docking results which suggest the mechanism of action of the anti-EV71 activity of this molecule. According to previous reports [30–32], a conformational change in the VP1 protein is crucial for viral particle disassembly and release of viral RNA into the host cell. To assess the potential effectiveness of RA in vivo, a neonatal ICR mouse model was used. Results showed that 20 mg/kg RA prolonged survival time and reduced mortality when mice were inoculated with a lethal dose of EV71 (Fig. 4a). The symptoms of EV71 infection were prevented by RA (Fig. 4b), and changes in body weight (Table 2) were consistent with the effects on morbidity and mortality.

In summary, we suggest that RA could affect the early stages (initial stage) of EV71 viral infection. It would be worth further evaluating the virucidal activity of RA in combination with other anti-EV71 inhibitors (data not published) for the effective treatment of EV71-induced HFMD in the future.

## Conclusions

The current study demonstrates that RA can effectively inhibit the early stage of EV71 viral infection both in vitro and in vivo. As such, RA could be developed as an antiviral agent against initial EV71 infection or as a synergistic therapeutic agent to prevent or reduce EV71-induced pathogenesis and complications.

## Data Availability

None.

## Abbreviations

ALD: a 3-(4-pyridyl)-2-imidazolidinone derivative with an amide at position 2 of the pyridine ring(molecular weight 476 Da)
CCID_50_: 50% cell culture infective dose
CPE: cytopathic effect
DAPI: 3,3'-diaminobenzidine
EV71: human enterovirus 71
HFMD: hand, foot, and mouth disease
MLB: magnesium lithospermate B
MTT: 3-(4,5-dimethylthiazol-2-yl)-2,5-diphenyltetrazolium bromide
NLD: a 3-(4-pyridyl)-2-imidazolidinone derivative with a primary amine at position 2 of the pyridine ring (molecular weight 448 Da)
PI: propidium iodide
RA: rosmarinic acid
RD cells: rhabdomyosarcoma cells
WIN 51711: 5-[7-[4-(4, 5-dihydro-2-oxazolyl) phenoxy] heptyl]-3-methylisoxazole

## References

[1] Chang PC, Chen SC, Chen KT (2016). The current status of the disease caused by enterovirus 71 infections: epidemiology, pathogenesis, molecular epidemiology, and vaccine development. Int J Environ Res Public Health.

[2] Yi L, Lu J, Kung HF, He ML (2011). The virology and developments towards control of human enterovirus 71. Crit Rev Microbiol.

[3] Wang L, Wang J, Wang L, Ma S, Liu Y (2015). Anti-enterovirus 71 agents of natural products. Molecules..

[4] Wang M, Tao L, Xu U (2016). Chinese herbal medicines as a source of molecules with anti-enterovirus 71 activity. Chin Med.

[5] Arita M, Wakita T, Shimizu H (2008). Characterization of pharmacologically active compounds that inhibit poliovirus and enterovirus 71 infectivity. J Gen Virol.

[6] Yang Y, Xiu J, Zhang L, Qin C, Liu J (2012). Antiviral activity of punicalagin toward human enterovirus 71 in vitro and in vivo. Phytomedicine..

[7] Yang Y, Xiu J, Liu J, Zhang L, Li X, Xu Y (2013). Chebulagic acid, a hydrolyzable tannin, exhibited antiviral activity in vitro and in vivo against human enterovirus 71. Int J Mol Sci.

[8] Chung YC, Hsieh FC, Lin YJ, Wu TY, Lin CW, Lin CT (2015). Magnesium lithospermate B and rosmarinic acid, two compounds presents in Salvia miltiorrhiza, have potent antiviral activity against enterovirus 71 infections. Eur J Pharmacol.

[9] Petersen M, Simmonds MS (2003). Rosmarinic acid. Phytochemistry..

[10] Amoah SKS, Sandjo LP, Kratz JM, Biavatti MW (2016). Rosmarinic acid-pharmaceutical and clinical aspects. Planta Med.

[11] Kim GD, Park YS, Jim YH, Park CS (2015). Production and applications of rosmarinic acid and structurally related compounds. Appl Microbiol Bioyechnol.

[12] Bulgakov VP, Inyushkina YV, Fedoreyov SA (2012). Rosmarinic acid and its derivatives: biotechnology and applications. Crit Rev Biotechnol.

[13] Tsukamoto Y, Ikeda S, Uwai K, Taguchi R, Chayama K, Sakaguchi T (2018). Rosmarinic acid is a novel inhibitor for hepatitis B virus replication targeting viral epsilon RNA-polymerase interaction. PLoS One.

[14] Chan YK, Sam IC, AbBakar S (2010). Phylogenetic designation of enterovirus 71 genotypes and subgenotypes using complete genome sequences. Infect Genet Evol.

[15] Dang DJ, Zhang C, Zhang RG, Wu WD, Chen SY, Ren JC (2017). Involvement of inducible nitric oxide synthase and mitochondrial dysfunction in the pathogenesis of enterovirus 71 infection. Oncotarget..

[16] Huang KYA, Chen MF, Huang YC, Shih SR, Chiu CH, Lin JJ, Wang JR (2017). Epitope-associated and specificity-focused features of EV71-neutralizing antibody repertoires from plasmablasts of infected children. Nat Commun.

[17] Yu P, Gao Z, Zhong YY, Bao LL, Xu L, Deng W (2014). Histopathological features and distribution of EV71 antigens and SCAB2 in human fatal cases and a mouse model of enterovirus 71 infection. Virus Res.

[18] Kroneman A, Vennema H, Deforche K, Avoort H, Peñaranda S (2011). An automated genotyping tool for enteroviruses and noroviruses. J Clin Virol.

[19] Qing J, Wang YX, Sun YN, Huang JY, Yan WZ, Wang JL (2014). Cyclophilin a associates with enterovirus-71 virus capsid and plays an essential role in viral infection as an uncoating regulator. PLoS Pathog.

[20] Lee H, Cifuene JO, Ashley RE, Conway JF, Makhov AM, Tano Y (2013). A strain-specific epitope of enterovirus 71 identified by cryo-electron microscopy of the complex with fab from neutralizing antibody. J Virol.

[21] Ye Xiaohua, Fan Chen, Ku Zhiqiang, Zuo Teng, Kong Liangliang, Zhang Chao, Shi Jinping, Liu Qingwei, Chen Tan, Zhang Yingyi, Jiang Wen, Zhang Linqi, Huang Zhong, Cong Yao (2016). Structural Basis for Recognition of Human Enterovirus 71 by a Bivalent Broadly Neutralizing Monoclonal Antibody. PLOS Pathogens.

[22] Wang X, Peng W, Ren J, Hu Z, Xu J, Lou Z (2012). A sensor-adaptor mechanism for enterovirus uncoating from structures of EV71. Nat Struct Mol Biol.

[23] Wallace AC, Laskowski RA, Thornton JM (1995). LIGPLOT: a program to generate schematic diagrams of protein-ligand interactions. Protein Eng.

[24] Wang YF, Yu CK (2014). Animal models of enterovirus 71 infection: applications and limitations. J Biomed Sci.

[25] Zhang W, Qiao H, Lv Y, Wang J, Chen X, Hou Y (2014). Apigenin inhibits enterovirus 71 infection by disrupting viral RNA association with trans-acting factors. PLoS One.

[26] Zhang L, Li Y, Gu Z, Wang Y, Shi M, Ji Y (2015). Resveratrol inhibits enterovirus 71 replication and pro-inflammatory cytokine secretion in rhabdosarcoma cells through blocking IKKs/NF-kappaB signaling pathway. PLoS One.

[27] Zhang H, Tao L, Fu WW, Liang S, Yang YF, Yuan QH (2014). Prenylated benzoylphloroglucinols and xanthones from the leaves of garcinia oblongifolia with antienteroviral activity. J Nat Prod.

[28] Choi HJ, Song JH, Park KS, Baek SH (2010). In vitro anti-enterovirus 71 activity of gallic acid from Woodfordia fruticosa flowers. Lett Appl Microbiol.

[29] Bemard A, Lacroxic C, Cabiddu MG, Neyts J, Leyssen P, Pompei R (2015). Exploration of the anti-enterovirus activity of a series of pleconaril/pirodavir-like compounds. Antivir Chem Chemother.

[30] De Colibus L, Wang X, Spyrou JAB, Kelly J, Ren J, Grimes J (2014). More-powerful virus inhibitors from structure-based analysis of HEV71 capsid-binding molecules. Nat Struct Mol Biol.

[31] Li P, Yu J, Hao F, He H, Shi X, Hu J (2017). Discovery of potent EV71 capsid inhibitors for treatment of HFMD. ACS Med Chem Lett.

[32] Plevka P, Perera R, Yap ML, Cardosa J, Kuhn RJ, Rossmann MG (2013). Structure of human enterovirus 71 in complex with a capsid-binding inhibitor. Proc Natl Acad Sci U S A.

